# A phase Ib dose-escalation and expansion study of the oral MEK inhibitor pimasertib and PI3K/MTOR inhibitor voxtalisib in patients with advanced solid tumours

**DOI:** 10.1038/s41416-018-0322-4

**Published:** 2018-11-14

**Authors:** Alison M. Schram, Leena Gandhi, Monica M. Mita, Lars Damstrup, Frank Campana, Manuel Hidalgo, Enrique Grande, David M. Hyman, Rebecca S. Heist

**Affiliations:** 1 Memorial Sloan Kettering Cancer Center, New York, NY USA; 2 New York University Perlmutter Cancer Center, New York, NY USA; 3 Cedars-Sinai Medical Center, Los Angeles, CA USA; 4 Merck Serono, Darmstadt, Germany; 5 Sanofi-Aventis, Cambridge, MA USA; 6 Beth Israel Deaconess Medical Center, Boston, MA USA; 7 Hospital Universitario Ramon y Cajal, Madrid, Spain; 8 Massachusetts General Hospital, Boston, MA USA

**Keywords:** Targeted therapies, Drug development

## Abstract

**Background:**

This phase Ib study evaluated the safety, maximum-tolerated dose (MTD), pharmacokinetics, pharmacodynamics, and preliminary efficacy of pimasertib (MSC1936369B), a MEK1/2 inhibitor, in combination with voxtalisib (SAR245409), a pan-PI3K and mTORC1/mTORC2 inhibitor, in patients with advanced solid tumours.

**Methods:**

This study included a dose escalation and expansion in patients with select tumour types and alterations in the MAPK or PI3K pathways. A 3 + 3 design was used to determine MTD. Patients were evaluated for adverse events and tumour response.

**Results:**

146 patients were treated, including 63 in dose escalation and 83 in expansion. The MTD was pimasertib 90 mg and voxtalisib 70 mg daily. Based on the safety profile, the recommended phase 2 dose (RP2D) was pimasertib 60 mg and voxtalisib 70 mg. The most frequent treatment-emergent adverse events (TEAEs) were diarrhoea (75%), fatigue (57%), and nausea (50%). Responses included a complete response in one patient (1%), partial response in five (5%), and stable disease in 51 (46%). At the RP2D, 74 patients required dose interruption (73%), 20 required dose reduction (20%), and 26 discontinued treatment due to TEAEs (26%).

**Conclusions:**

The combination of pimasertib and voxtalisib showed poor long-term tolerability and limited anti-tumour activity in patients with advanced solid tumours.

## Introduction

The MAPK (RAS/RAF/MEK/ERK) and PI3K/mTOR signalling pathways are among the most frequently altered in cancer, where they are important contributors to tumour growth and survival.^1,2^ Activating *RAS* and *BRAF* mutations are seen in approximately 15 and 8% of all cancers, respectively.^3,4^ *KRAS* mutations are common in several solid tumours including pancreatic cancer, colorectal cancer (CRC), and non-small cell lung cancer (NSCLC),^5^ while *BRAF* mutations are prevalent in melanoma.^6^ Aberrant activation of the PI3K pathway typically occurs through activating mutations in *PIK3CA* or by loss-of-function or epigenetic silencing of *PTEN*.

The PI3K and MAPK pathways are closely linked by feedback loops leading to compensatory activation of one in response to inhibition of the other.^7^ In patients, concurrent genomic alterations and/or upregulation in components of both pathways are frequently observed. In vitro and in vivo preclinical models have demonstrated enhanced anti-tumour effects when both pathways are targeted simultaneously.^8^ Therefore, combination therapy with drugs targeting the MAPK and PI3K signalling pathways is predicted to improve efficacy compared with inhibition of either cascade alone and may be an effective therapeutic strategy in the treatment of a variety of cancers.

Pimasertib (MSC1936369B, formerly known as AS703026) is a potent and selective, ATP non-competitive small molecule inhibitor of MEK1 and MEK2 that has shown efficacy in preclinical models.^8^ In a phase I, first-in-human study of this compound, the adverse event profile of pimasertib was consistent with other agents in this class and include skin rash, diarrhoea, asthenia, anorexia, nausea/ vomiting, peripheral oedema, anaemia and visual disorders, including serous retinal detachment and retinal vein occlusion. Two dosing schedules were explored, including Schedule 1 consisting of dosing on days 1–5, 8–12, and 15–19, and Schedule 2 consisting of dosing on days 1-15 of a 21-day cycle. The maximum tolerated doses (MTDs) were 120 mg per day and 195 mg per day, respectively.^9^

Voxtalisib (SAR245409, formerly known as XL765) is a highly selective and potent, ATP-competitive, reversible dual pan-Class I PI3K and mTORC1/mTORC2 inhibitor.^10–12^ The phase I, first-in-human study of this compound demonstrated a manageable safety profile, reduced PI3K and mTORC1/mTORC2 pathway signalling, and was associated with clinically relevant stable disease. The most frequent treatment-related adverse events were nausea, diarrhoea, vomiting, and decreased appetite. Both once and twice daily schedules were explored. The MTDs were 90 mg once daily and 50 mg twice daily.^13^

The aim of this phase Ib study was to determine MTD and recommended phase 2 dose (RP2D) for pimasertib combined with voxtalisib when administered orally to patients with selected advanced solid tumours. Additional objectives included evaluating safety and preliminary antitumour activity (clinicaltrials.gov registry identifier NCT01390818).

## Materials and methods

This study was conducted across 10 centres in the United States and three centres in Europe. It was performed in accordance with the Declaration of Helsinki and the International Conference on Harmonisation Good Clinical Practice guidelines.

### Patient population

Eligible patients were ≥ 18 years old with a histologically or cytologically confirmed diagnosis of an advanced solid tumour (pancreatic, thyroid, colorectal, non-small cell lung, endometrial, renal, breast, ovarian carcinoma, or melanoma) and/or any advanced solid tumour with an alteration in one or more of the following genes: *PTEN*, *BRAF*, *KRAS*, *NRAS*, *PI3KCA*, *EGFR*, *ERBB2*, *MET*, *RET*, *c-KIT*, *GNAQ*, *GNA11*. Subjects were also required to have archived tumour tissue available for central analysis, measurable disease by Response Evaluation Criteria in Solid Tumours (RECIST) v1.1, Eastern Cooperative Group Oncology (ECOG) performance score of 0 to 1, and adequate organ and bone marrow function. Patients were excluded if they had a history of central nervous system metastases (unless documented as stable by imaging), ocular/retinal comorbidities associated with increased risk of central serous retinopathy or retinal vein occlusion, previous anti-cancer therapy within 28 days of the first infusion or within five half-lives of treatment (whichever was shorter), or recent major surgery or trauma. Patients who had been treated with a PI3K or MEK inhibitor were excluded if they had been discontinued due to treatment-related adverse events (AEs). Following dose escalation, disease specific expansion cohorts were initiated in patients with *KRAS* or *NRAS*-mutant NSCLC, triple negative breast cancer (TNBC), dual *KRAS* and *PIK3CA*-mutant CRC, or *BRAF* V600-mutant melanoma that had progressed on BRAF inhibitors.

### Study design and treatment

This was a phase Ib, open-label, nonrandomised, dose-escalation and dose-expansion study. The primary endpoint was the incidence of dose limiting toxicities (DLTs) and determination of the MTD and RP2D. Secondary endpoints included tumour response as assessed by RECIST v1.1, characterisation of treatment-emergent adverse events (TEAEs), pharmacokinetics (PK), treatment-mediated changes in the MAPK and PI3K pathways (pERK and pS6 levels in blood and tumour biopsies), and correlation of MAPK and PI3K pathway alterations with response to therapy.

**Fig. 1:**
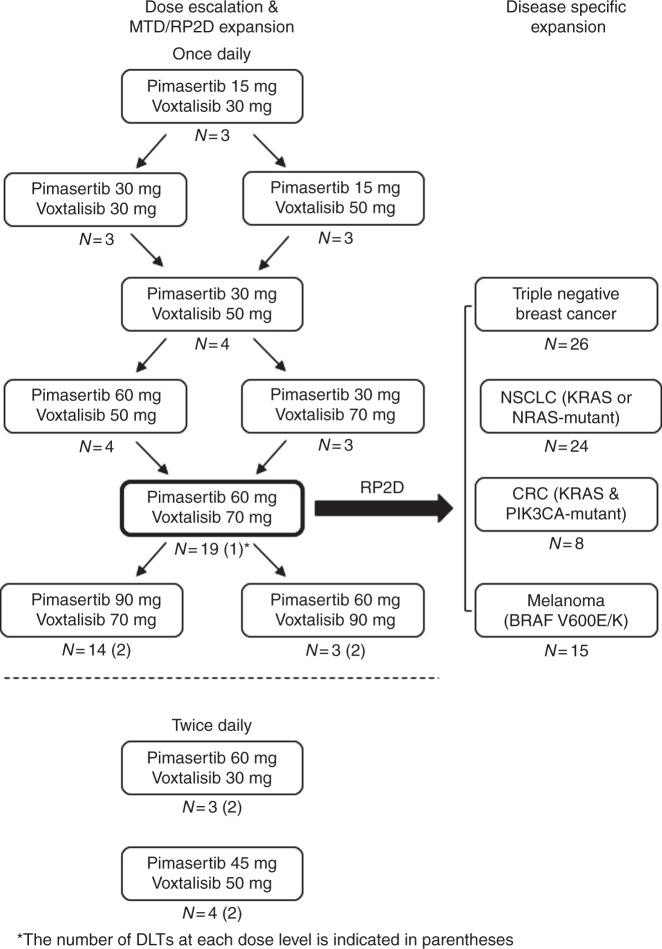
Dose-escalation schedule

A modified “3 + 3” dose escalation design was followed with once daily dosing (Fig. 1). The MTD was defined as the highest dose with 0 out of 3 patients or ≤1 out of 6 patients with a DLT. Once an MTD was reached, additional subjects were enroled in an MTD dose expansion to confirm the tolerability of this dose level. Additional subjects could also be enroled at lower dose levels (LDLs) at the discretion of the safety monitoring committee consisting of members from the sponsor and investigator. After completion of the once daily dosing dose escalation, twice daily dosing was explored whereby the monotherapy RP2D of each drug was combined with a lower dose of the combination partner in 2 parallel cohorts. The final RP2D of the combination was defined by the safety monitoring committee based on the totality of safety and tolerability data generated during the dose escalation phase of the study. After selection of the RP2D of the combination, four tumour-specific expansion cohorts were opened for patients with advanced NSCLC, TNBC, CRC, and melanoma as explained above.

A subset of patients were evaluated for drug-drug interactions consisting of a four days run-in prior to cycle 1 day 1 during which each compound was administered separately. All subjects were treated in 21-day treatment cycles until disease progression, intolerable toxicity, or withdrawal. Both pimasertib and voxtalisib were orally administered and taken together, in a fasted state, continuously in once daily or twice daily dosing schedules.

A DLT was defined as a grade ≥3 adverse event attributed as at least possibly related to a study medication by investigators or any adverse that resulted in a treatment delay of >2 weeks. The DLTs were monitored for during cycle 1. Safety assessments were performed throughout the study treatment period and included vital sign measurements, physical examinations, clinical laboratory tests, 12-lead electrocardiogram, echocardiogram or MUGA scan, urinalysis, and collection of AE information. An ophthalmologic exam was performed at screening and every other cycle. This included funduscopy, a slit lamp examination, intraocular pressure measurement, visual acuity and field testing, and optical coherence tomography. Adverse events were graded using NCI Common Toxicity Criteria of Adverse Events (CTCAE) version 4.0.^14^ Radiologic assessments of tumour response by computed tomography or magnetic resonance imaging were conducted at baseline and every other cycle thereafter according to RECIST v1.1.^15^

### Pharmacokinetic (PK) assessments

Blood samples for serial PK evaluation for pimasertib and voxtalisib were collected after concomitant administration of a single dose on Day 1 and after multiple doses on Day 15 in Cycle 1 of all cohorts. Blood samples for serial PK evaluation for pimasertib and voxtalisib were collected after concomitant administration of a single dose on Day 1 and after multiple doses on Day 15 in Cycle 1 of all cohorts at the following time points: pre-dose, 0.5, 1, 1.5, 2, 3, 4, 8, 10, and 24 h. The 10-hour time point was omitted in the disease specific dose expansion cohorts. In addition, the PK of pimasertib and voxtalisib were determined for both compounds dosed separately in a drug-drug evaluation period prior to Cycle 1.

### Pharmacodynamic and biomarker assessment

In order to assess the on-target activity of pimasertib and voxtalisib, peripheral blood mononuclear cells (PBMC) were collected at prespecified time points in patients treated in the dose escalation. Levels of intracellular pERK (T202/Y204) and pS6 (S240/S244) were assessed by flow cytometry. Tumour biopsies taken pre-treatment and on cycle 1, day 19 ± 1 day were evaluated for pERK (T202/Y204) and pS6 (S240/S244). If available, fresh or archival tissue was tested for mutations in *KRAS*, *NRAS*, *BRAF*, and *PIK3CA*. Plasma samples were taken at baseline and sequenced for these same genes.

### Statistical analysis

The sample size of the dose escalation cohort was determined by the tolerability of the combination according to a classical 3 + 3 design. The sample size of the expansion cohorts was based on the hypothesis that a sample size of 18 evaluable patients was sufficient to reject the null hypothesis (RR ≤ 10%) when the true response rate is ≥30%, with a power of 80%. Safety and efficacy data were reported using descriptive statistics and were based on the database lock date of July 1st, 2015. For categorical variables, frequencies and percentages were employed. Continuous variables were described using the mean, standard deviation, median, range, and number of missing values. The response rate (RR) and disease control rate (DCR) with the respective 2-sided 90% exact confidence intervals (CIs) were computed.

Pharmacokinetic parameters were derived using the non-compartmental analysis module in Phoenix WinNonlin (Version 6.4) and were summarised using descriptive statistics. Peak plasma concentrations (C_max_) and time for the peak plasma concentrations (T_max_) were the observed values. The areas under the concentration-time curve (AUC_0-t_) were calculated using the linear trapezoidal rule. The terminal elimination rate constant (λz) was determined by regression analysis of the linear terminal portion of the log plasma concentration-time curve. Graphics were prepared with SAS (Version 9.2) and SigmaPlot (Version 12.5).

Pharmacokinetic drug-drug interactions (DDI) between pimasertib and voxtalisib were assessed by comparing the ratios of each drug’s AUC0-t and Cmax on Day 1 of Cycle 1 to Day 1 of the DDI evaluation period. These comparisons were made by dose level using a linear mixed model with a fixed effect for treatment and a random effect for subject on the log-transformed values of AUC0-t and Cmax on Day 1 of Cycle 1. Geometric least squares means with corresponding 95% CIs per treatment, and ratios of geometric least squares means with corresponding 90% CIs for test/reference were estimated by exponentiation of estimates from the mixed model.

## Results

### Patients

A total of 146 patients were enroled and treated on this study, including 63 in the dose escalation and 83 in the dose expansion. The majority of patients were female (*n* = 87, 60%). Patient age ranged from 26 to 82 years old. The most common cancer types were CRC (*n* = 37, 25%), NSCLC (*n* = 33, 23%), breast (*n* = 28, 19%), and melanoma (*n* = 20, 14%). Baseline patient characteristics are summarised in Table 1. All 146 patients came off treatment during the trial. The primary reason for discontinuation was progressive disease (*n* = 105, 72%), followed by adverse events (*n* = 22, 15%), withdrawal (*n* = 13, 9%), and other (*n* = 6, 4%).

**Table 1:** Baseline characteristics of patients enroled

Characteristics	Cohorts		
	Once daily dose escalation (*N* = 56)	Twice daily dose escalation (*N* = 7)	Disease specific expansion (*N* = 83)
Gender, *n* (%)			
Male	27 (48.2)	5 (71.4)	27 (32.5)
Female	29 (51.8)	2 (28.6)	56 (675)
Age (years)			
Median (range)	58.0 (26–82)	58.0 (26–70)	59.0 (27–80)
Race, *n* (%)			
White	51 (91.1)	7 (100.0)	75 (90.4)
Black/African American	5 (8.9)	0	2 (2.4)
Asian	0	0	5 (6.0)
Other	0	0	1 (1.2)
ECOG PS, *n* (%)			
0	25 (44.6)	4 (57.1)	35 (42.2)
1	30 (53.6)	3 (42.9)	47 (56.6)
≥2	1 (1.8)	0	1 (1.2)
Tumour types, *n* (%)			
Breast	3 (5.4)	0	25 (30.1)
Colorectal	16 (28.6)	3 (42.9)	18 (21.7)
Melanoma	3 (5.4)	0	17 (20.4)
Non-small cell lung	8 (14.3)	2 (28.6)	23 (25.3)
Ovarian	11 (19.6)	1 (14.3)	0
Pancreas	7 (12.5)	0	0
Other	8 (14.3)	1 (14.3)	0

### Safety and tolerability

Eleven dose levels were explored during the dose escalation in two schedules: daily administration (pimasertib 15–90 mg and voxtalisib 30–90 mg, *n* = 9 dose levels) and twice daily administration (pimasertib 45–60 mg and voxtalisib 30–50 mg, *n* = 2 dose levels) (Fig. 1).

Nine patients in dose escalation experienced a DLT during cycle 1, including five patients dosed once daily and four patients dosed twice daily. These DLTs included grade 3 nausea (2 subjects), grade 3 vomiting, grade 3 AST increase, grade 3 serous retinal detachment (SRD) in the context of vision changes, grade 3 fatigue, and grade 3 rash maculopapular (3 subjects). The MTD was established as pimasertib 90 mg and voxtalisib 70 mg once daily. Based on the overall safety profile, a RP2D of pimasertib 60 mg and voxtalisib 70 mg was selected for dose expansion. Twice daily dosing was abandoned prior to establishing an MTD due to unacceptable toxicity, exemplified by DLTs in 2 out of 3, and 2 out of 4 patients studied at the initial dose levels (Fig. 1). At the RP2D, 74 patients required at least one dose interruption (73%), 20 required a dose reduction (20%), and 26 discontinued the study due to TEAEs (26%).

The most common TEAEs (those occurring in ≥20% of subjects) were diarrhoea, fatigue, nausea/vomiting, and acneiform dermatitis (Table 2). The most commonly reported grade ≥3 TEAEs (occurring ≥10% of subjects) were hyponatremia (13%), disease progression (13%), hypokalaemia (11%), and maculopapular rash (10%) in the dose escalation, and fatigue (11%) in the disease-specific expansion. Twenty-one (14%) subjects had a serious adverse event possibly related to the study drugs, most commonly fever (3%), nausea (3%), dehydration (3%), and diarrhoea (2%). The study drugs were felt to be a contributing factor in the death of one patient who died of pancreatitis and disease progression.

**Table 2:** Treatment-emergent adverse events (occurring in ≥20% of subjects)

Adverse event, *N* (%)	Dose escalation (*N* = 63)		Dose expansion (*N* = 83)		All (*N* = 146)	
	Grades					
	All	≥3	All	≥3	All	≥3
Diarrhoea	45 (71)	4 (6)	64 (77)	6 (7)	109 (75)	10 (7)
Fatigue	39 (62)	4 (6)	44 (53)	9 (11)	83 (57)	13 (9)
Nausea	31 (49)	4 (6)	42 (51)	3 (4)	73 (50)	7 (5)
Vomiting	31 (49)	2 (3)	39 (47)	3 (4)	70 (48)	5 (3)
Dermatitis acneiform	25 (40)	6 (10)	32 (39)	1 (1)	57 (39)	7 (5)
Maculopapular rash	24 (38)	6 (10)	27 (33)	6 (7)	51 (35)	12 (8)
Peripheral edema	26 (41)	1 (2)	19 (23)	0 (0)	45 (31)	1 (1)
Pyrexia	24 (38)	0 (0)	21 (25)	1 (1)	45 (31)	1 (1)
Decreased appetite	17 (27)	0 (0)	25 (30)	1 (1)	42 (29)	1 (1)
Stomatitis	11 (18)	0 (0)	23 (28)	5 (6)	33 (23)	5 (3)
Dyspnoea	12 (19)	3 (5)	17 (21)	5 (6)	29 (20)	8 (6)

Adverse events of special interest including ocular toxicity and ejection fraction decrease. A total of 47 subjects (32%) had ocular adverse events, including focal retinal detachment in 27 (19%), macular detachment in 15 (10%), detachment of the retinal pigment epithelium in 7 (5%), macular oedema in 2 (1%), and chorioretinopathy in 1 (1%). No subject experienced retinal vein occlusion. Ejection fraction decreases of ≥10% were observed in 39 (26.7%) patients (29 grade 2, 10 grade 3) across all dose levels and schedules. Rates were roughly equivalent across the dose escalation and expansion cohorts. None of these ejection fraction decreases were deemed by investigators to be serious adverse events. Both ocular and cardiac toxicity were reversible.

### Efficacy

**Fig. 2:**
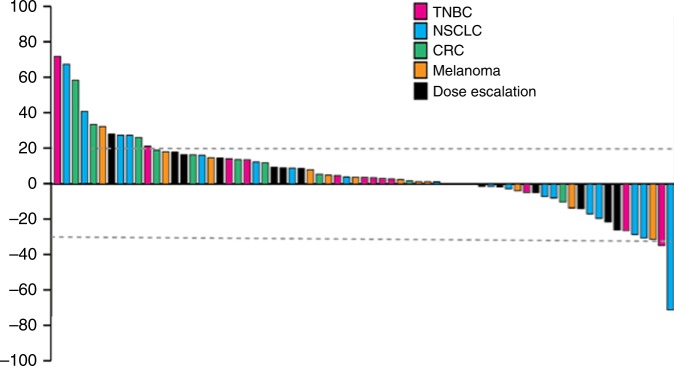
Maximum change in tumour size at the RP2D, according to tumour type

Among the 146 treated subjects, 110 patients had a baseline and at least one post baseline radiologic assessment and were therefore included in the efficacy analysis set. The best overall responses included a complete response (CR) in one patient (1%), partial response (PR) in five patients (5%), stable disease (SD) in 51 patients (46%), and progressive disease (PD) in 45 patients (41%). The CR occurred in a patient with melanoma treated in the disease specific expansion cohort. The five subjects with PRs included three in the dose escalation cohorts dosed once daily, and two subjects in the disease specific expansion cohorts (one with NSCLC and one with melanoma). Therefore, the response rate was 14% in the melanoma cohort (2/14 evaluable patients), 5% in the NSCLC cohort (1/20), and 0% in the TNBC and CRC cohorts (0/16 and 0/11, respectively). Notably, two responders in the dose escalation cohort had mutations in the MAPK or PI3K pathways, including one with KRAS-mutant CRC with neuroendocrine features and another with dual KRAS/PIK3CA-mutant low grade serous ovarian cancer. Best tumour responses in patients treated at the RP2D are shown (Fig. 2). The median time on treatment was 2 cycles (6 weeks). Recruitment into the CRC and melanoma cohorts was stopped early due to the absence of early signs of clinical efficacy.

### Pharmacokinetics

Pimasertib and voxtalisib were rapidly absorbed following oral administration (median Tmax approximately 1–2 h). Exposure generally increased with an increase in dose for both pimasertib and voxtalisib, appearing less than dose proportional for pimasertib and close to proportional for voxtalisib in the daily regimens (Supplementary Fig. S1–2). The half-life using daily dosing was similar across groups in the dose escalation, ranging from 4.3 h to 6.1 h on Day 1 and 5.5 to 7.2 h on Day 15 for pimasertib and 3.1 to 3.6 h on Day 1 and 3.4 to 5.2 h on Day 15 for voxtalisib. There was variable accumulation across doses by Day 15 following once daily dosing with pimasertib and no apparent accumulation with voxtalisib. Pimasertib administration decreased voxtalisib exposure (AUC_0-t_) by 11% on average (90% CI of 67.0 to 118.8) and voxtalisib administration increased pimasertib exposure (AUC_0-t_) up to 49% on average (90% CI of 106.2 to 208.9).

### Pharmacodynamics and biomarker analysis

Peripheral blood mononuclear cells were analysed for pERK and pS6 in nine subjects, all of whom were in the once daily dose escalation cohort and with no more than one patient representing each dose level. In the patient dosed at the RP2D, near complete inhibition was observed for both pERK and pS6 across most of the dosing interval. The biomarker analysis set for predictive genomic markers (*KRAS*, *NRAS*, *BRAF*, or *PIK3CA* mutations) included 46 subjects with tumour tissue sequenced. Given the low number of subjects with an observed objective response (CR or PR), no conclusion could be drawn in terms of the correlation between clinical activity and mutational profile.

## Discussion

This phase 1b study demonstrates that the MTD of the oral MEK1/2 inhibitor, pimasertib, and oral PI3K and mTORC1/2 inhibitor, voxtalisib, is pimasertib 90 mg and voxtalisib 70 mg once daily. Based on the overall safety profile, the RP2D was chosen as one dose level below the MTD, or pimasertib 60 mg and voxtalisib 70 mg. Despite choosing this lower dose for evaluation in the expansion cohorts, the majority of patients treated at this dose required treatment interruptions, dose reductions, or drug discontinuation due to chronic toxicity, suggesting that the overall tolerability of the combination may be challenging. The twice daily dosing schedule was abandoned due to poor tolerability.

The safety profile of this combination was consistent with that of other regimens that have attempted to target both the MAPK and PI3K/mTOR pathways simultaneously.^16–19^ For example, in a phase Ib study of the oral pan-PI3K inhibitor, buparlisib, in combination with the oral MEK1/2 inhibitor, trametinib, the most common adverse events included elevated creatinine kinase, diarrhoea, nausea, stomatitis, and rash.^16^ Long-term tolerability was challenging and both dose interruptions and reductions were common. The significant long-term toxicity observed with strategies that combine MAPK and PI3K/mTOR pathway inhibition is likely due to several factors. These classes of agents have some overlapping toxicity that includes fatigue, anorexia, nausea/vomiting, diarrhoea, and rash. It is noteworthy that many of the preclinical studies evaluating the efficacy of this combination have been done in model systems such as mice that historically have tolerated inhibitors of the MAPK pathway better than humans. In addition, therapeutic agents targeting these pathways have historically not had selectively for mutant over wild-type kinases. Given that both pathways play important biological roles in many host tissues, it is perhaps expected that the combination presents long-term tolerability concerns. This trial exemplifies how defining DLTs only in cycle 1 may be insufficient to accurately characterise the tolerability of therapy, especially when studying continually-dosed targeted drugs. Cumulative toxicity and intolerability of chronic low-grade symptoms such as fatigue and nausea are not effectively captured using the standard trial design that only considers cycle 1 when defining the MTD. The frequency of dose reductions, dose holds, and general toxicity in later cycles must be considered when ultimately deciding the RP2D.

Despite preclinical data suggesting that the combination of pimasertib and voxtalisib would be synergistic, the clinical efficacy was limited and does not support this as a promising treatment option. Although it is possible that the limited dataset and patient heterogeneity contributed to the lack of observed efficacy, it is likely that the absence of cancer cell selectivity and narrow, or in some cases absent, therapeutic index associated with inhibiting these critical cellular pathways underlies the lack of clinical activity when this combination is administered at tolerable doses. Intermittent dosing may have achieved a more favourable therapeutic index, however, this theory has not borne out in similar trials testing PI3K/mTOR and MEK inhibitor combinations.^17,19^ The phase Ib study of buparlisib and trametinib demonstrated minimal activity in NSCLC and pancreatic cancer. Intriguingly, the response rate was 29% in patients with ovarian cancer with a signal of increased efficacy in patients with KRAS-mutant tumours.^16^ Similarly, a phase 1b study of binimetinib (MEK inhibitor) with apelisib (alpha-specific PI3K inhibitor), and another phase I study of gedatolisib (PI3K/mTOR inhibitor) in combination with PD-0325901 (MEK inhibitor) also showed tumour responses in KRAS-mutant ovarian cancers.^17^ The combination of pimasertib and voxtalisib was explored in a randomised phase II trial of women with low grade ovarian cancer (NCT01936363) but the study was terminated approximately halfway through accrual and the combination is not undergoing active development for this indication.

This study establishes that poor long-term tolerability prevents the combination of pimasertib and voxtalisib from being a meaningful therapeutic option at the tested dosing schedule. Future studies of agents targeting the MAPK and PI3K/mTOR pathway should include alternative dosing schedules and further explore the relationship between tumour type, genomic alterations, and efficacy.

## Electronic supplementary material

Supplemental Figure

## **Data availability:**

Available through EMD Serono.
