# Supplementary material for: A phase Ib dose-escalation and expansion study of the oral MEK inhibitor pimasertib and PI3K/MTOR inhibitor voxtalisib in patients with advanced solid tumours
Source: Br J Cancer. 2018 Nov 14;119(12):1471–6. doi: 10.1038/s41416-018-0322-4 (PMC6288157; doi:10.1038/s41416-018-0322-4)

**Supplementary Figures**

**Supplementary Figure 1.** Mean concentration-time profiles for pimasertib and voxtalisib (dose-escalation, daily dosing)


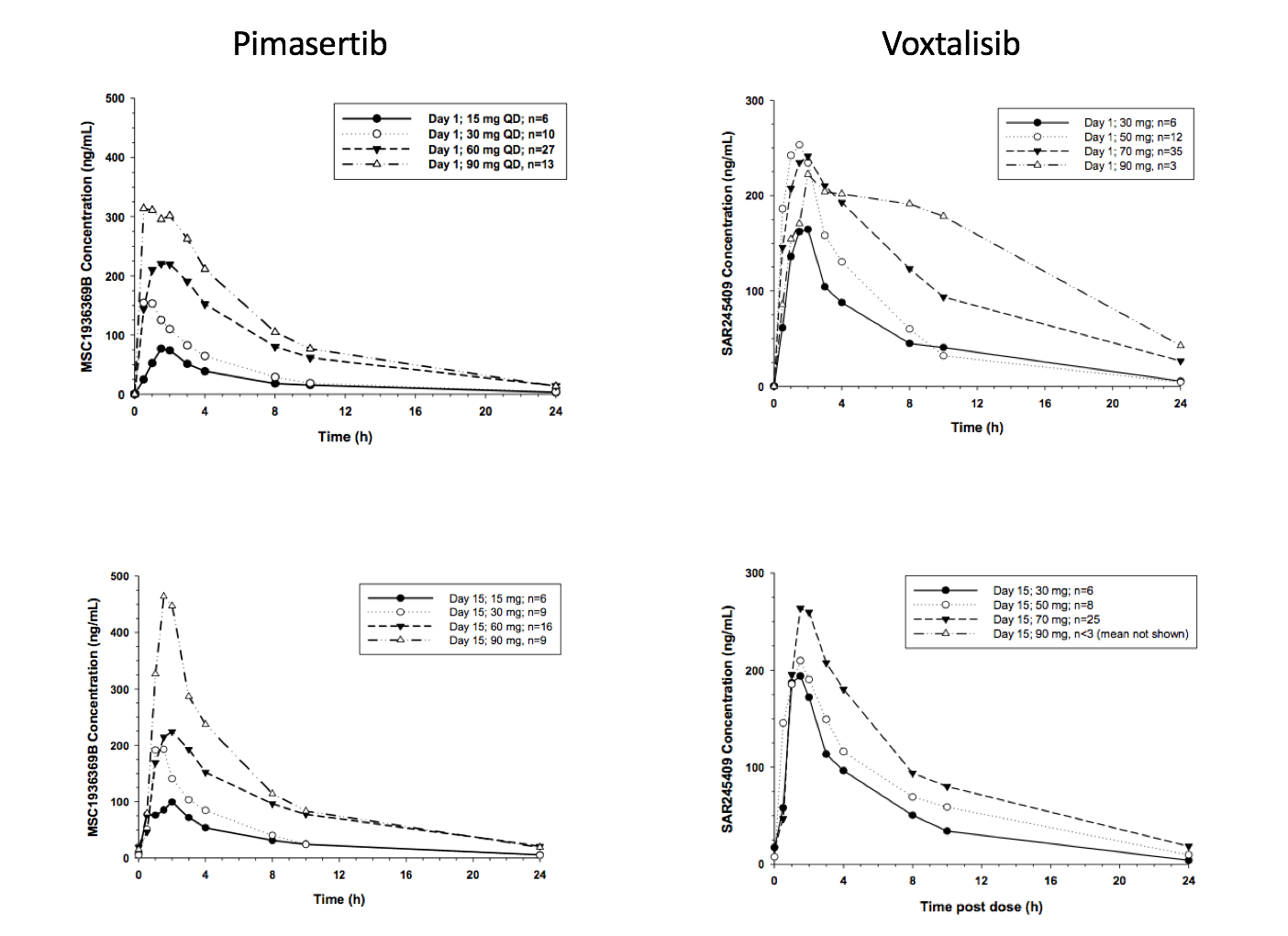


**Supplementary Figure 2.** Mean concentration-time profiles for pimasertib and voxtalisib (dose-escalation, twice daily dosing)


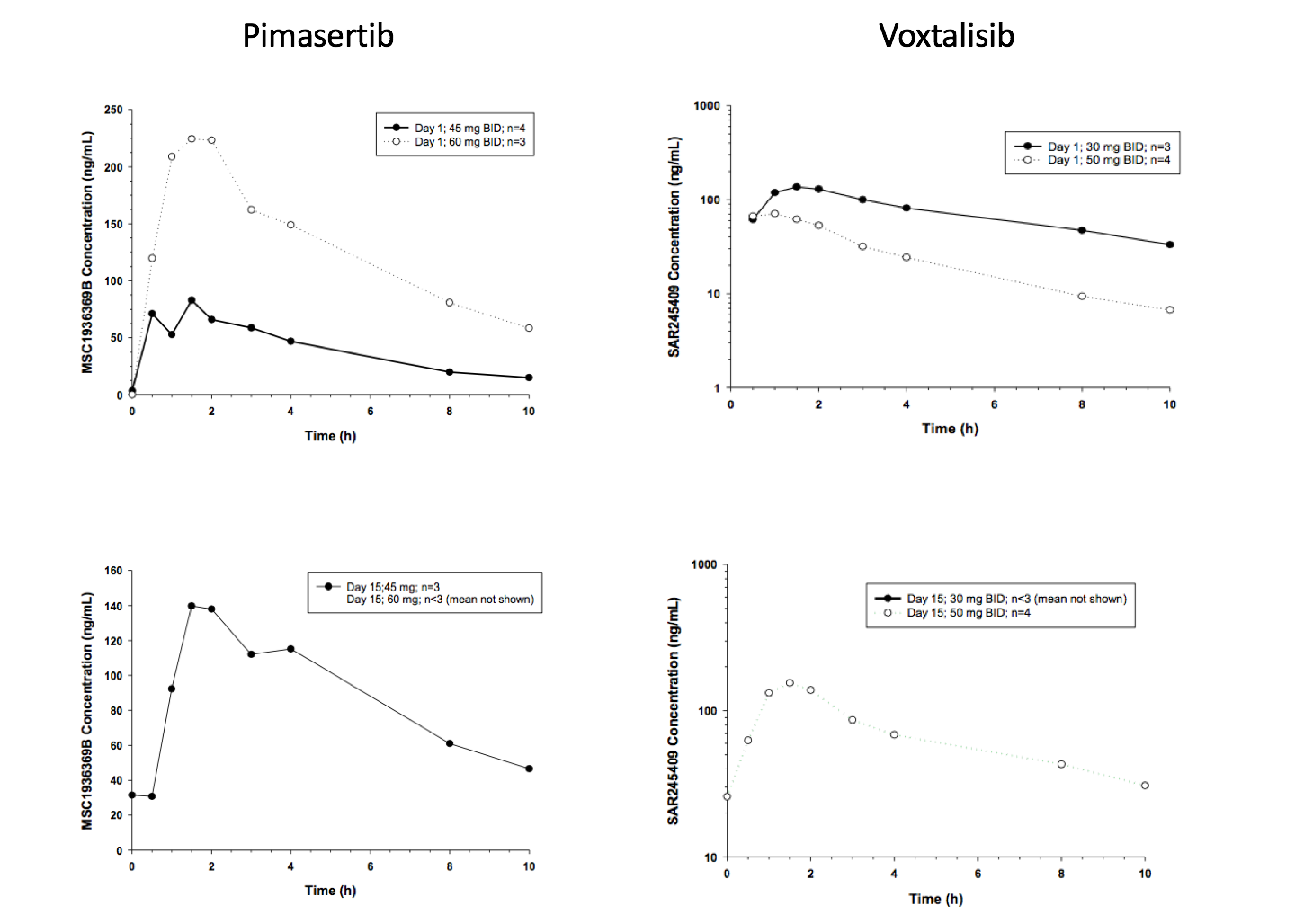

Supplement: Supplementary file 1 — Supplemental Figure [file 41416_2018_322_MOESM1_ESM.docx]
